# Size-based detection of sarcoma circulating tumor cells and cell clusters

**DOI:** 10.18632/oncotarget.20697

**Published:** 2017-08-24

**Authors:** Masanori Hayashi, Peixuan Zhu, Gregory McCarty, Christian F. Meyer, Christine A. Pratilas, Adam Levin, Carol D. Morris, Catherine M. Albert, Kyle W. Jackson, Cha-Mei Tang, David M. Loeb

**Affiliations:** ^1^ Sidney Kimmel Comprehensive Cancer Center, Johns Hopkins University, Baltimore, MD, USA; ^2^ Creatv MicroTech, Inc., Rockville, MD, USA; ^3^ Department of Orthopaedic Surgery, Johns Hopkins University School of Medicine, Baltimore, MD, USA; ^4^ Current affiliation: Seattle Children’s Hospital, University of Washington, Seattle, WA, USA

**Keywords:** biomarker, neoplastic cells, circulating, animal models, xenograft

## Abstract

Metastatic disease is the most important factor in determining the survival of sarcoma patients. Since sarcoma metastasis is predominantly hematogenous, we hypothesized that detection and quantification of circulating tumor cells (CTCs) could reflect response to therapy and risk of metastatic relapse. We evaluated the presence of CTCs using a novel animal model and in the blood of patients with high grade sarcomas utilizing the CellSieve™ size-based low pressure microfiltration system. Sarcoma CTCs were identified based on antibody staining patterns and nuclear morphology. Additionally, RNA was extracted from the CTCs for molecular analysis including demonstration of an EWS-FLI1 translocation, identification of a previously unrecognized p53 mutation in a patient with Ewing sarcoma, and single cell RNA sequencing of CTC from a child with alveolar rhabdomyosarcoma. In mouse xenograft models, the presence of CTC correlates with disease burden and with clinically silent metastases. In human patients, CTCs were readily detected at diagnosis, decreased with successful treatment, and were detectable in the blood of patients with no radiographic evidence of disease prior to the development of overt metastasis. Although evaluation of CTC is established in the care of patients with carcinomas, this technology has yet to be effectively applied to the evaluation and treatment of sarcoma patients. Our work demonstrates that the CellSieve™ microfiltration system can be used to study the biology of CTC in both mouse models and human sarcoma patients, with the potential for application to the monitoring of disease response and prediction of metastatic relapse.

## INTRODUCTION

Metastatic disease is the most common cause of death in patients with high-grade sarcoma. Despite effective local control in virtually all such patients, only a very small subset will avoid the development of distant metastasis and survive long term with surgical resection or radiation alone [1, 2]. With the introduction of neoadjuvant and adjuvant chemotherapy, long term survival for patients with localized disease has dramatically improved, ranging up to 70%, supporting the presence of micrometastatic disease (and presumably circulating tumor cells) at diagnosis in most patients [3, 4]. In contrast, only 10-20% of patients who present with advanced stage disease are cured with conventional chemotherapy [5, 6]. Intensification of chemotherapy has not substantially improved survival, but has resulted in significant morbidity in many patients [7]. The ability to quantify, isolate, and study circulating tumor cells (CTCs), the presumed agents of hematogenous metastasis, would improve our ability to monitor disease response to treatment, predict metastatic recurrence, and develop new therapies based on a deeper understanding of the biology of metastasis.

Although quantifying CTCs in carcinoma patients is an established element of care [8], there have been very few studies of CTCs in sarcoma patients. Use of flow cytometry to identify sarcoma CTCs, based on expression of either CD99 or cell surface vimentin, has been reported [9-11]. The use of CD99 is limited by background expression on normal hematopoietic cells [12], and also by being somewhat specific for Ewing sarcoma. Cell surface vimentin appears to be a more specific marker of sarcoma, not hematopoietic, cells, and is expressed on nearly all histologic subtypes of sarcoma [10]; however, the use of flow cytometry requires extensive processing of cells, which might affect their biology, and gating on single cells eliminates the possibility of detecting CTC clusters, which have been reported to be metastatic precursors in breast cancer [13, 14]. In addition, these and other positive-selection methods based on antibody capture suffer the same major drawback—the heterogeneous expression of target markers on CTC can result in variable sensitivity. CellSieve™ is a low pressure microfiltration device created by Creatv MicroTech which has been shown to be an effective method to efficiently collect CTCs in a variety of carcinomas [15]. This approach has several potential benefits, including minimal cell processing and the lack of a need for a sarcoma-specific cell surface antigen. We report here our results using this technology to collect, quantify, and characterize at a molecular level CTCs and CTC clusters in both preclinical models of sarcoma metastasis and in patients with high grade sarcomas. We also present proof of principle analyses suggesting that the presence of CTCs in patients with no radiographic evidence of disease predicts imminent relapse. Ours is the first report of a microfiltration system for the collection of sarcoma CTCs, is the largest report thus far, using any technology, of CTC quantification in sarcoma patients, and is the first report of molecular analysis of sarcoma CTCs.

## RESULTS

### *In vitro* assay validation

**Figure 1:**
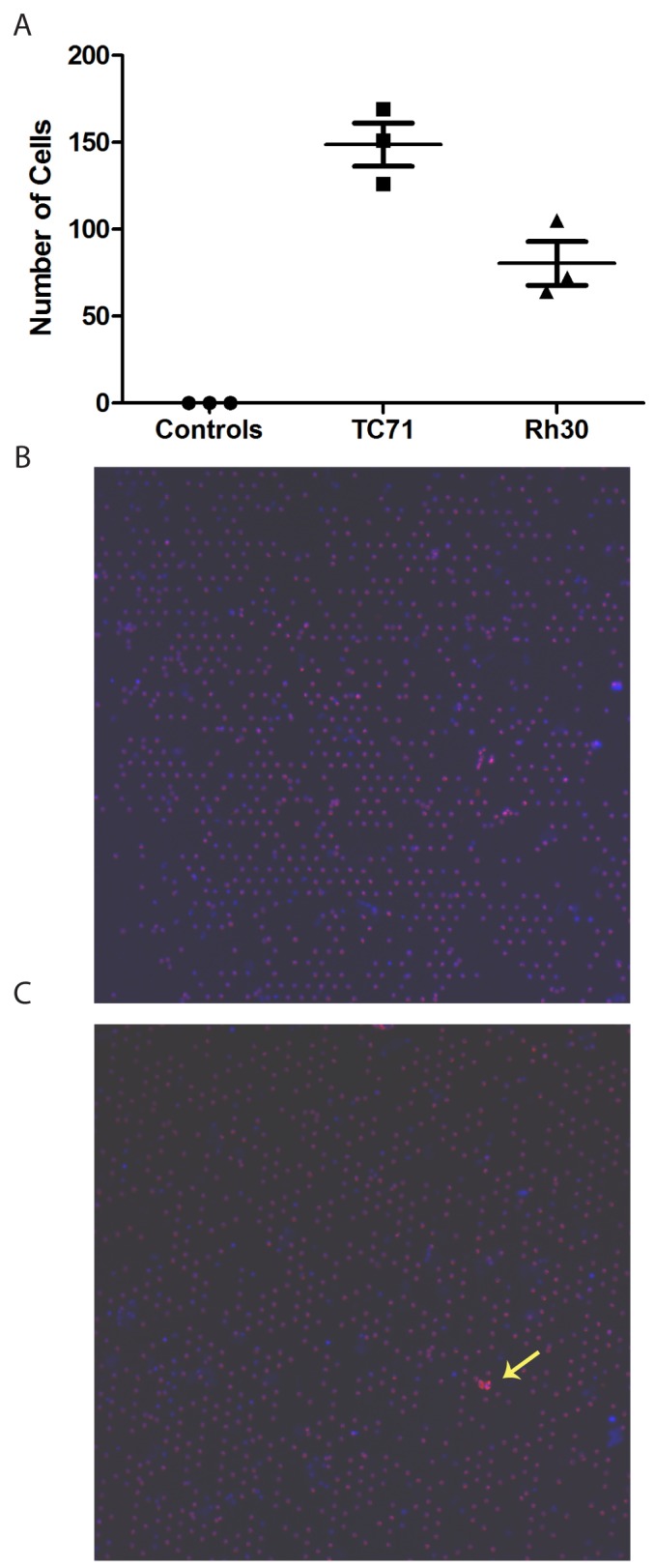
Validation of methodology **(A)** Blood from healthy volunteers was filtered (control) or was spiked with approximately 100 cells from the indicated cell line and then filtered. Membranes were stained as described and the number of TdTomato-positive, CD45-negative, vimentin-positive cells was quantified on each membrane. **(B)** Representative low power (10x) image of filtered normal blood. **(C)** Representative low power (10x) image of blood spiked with TdTomato-labeled TC71 cells (yellow arrow).

To validate the quantitative recovery efficiency of the CellSieve™ system for sarcoma cells, we performed a tumor cell spiking experiment with TC71 and RH30 cells. A cell suspension at concentration of 10,000 cells/ml was prepared, and 10 μL (approximately 100 live tumor cells) was spiked into 6 ml of whole blood from a healthy donor. The blood samples were immediately filtered through a CellSieve^™^ membrane, and the number of sarcoma cells retrieved was enumerated (Figure 1). As a negative control, blood from 3 healthy volunteers was also filtered and evaluated in parallel. All spiking experiments were carried out in triplicate. We recovered a mean of 148.7±21.6 TC71 cells and 80.3±21.7 RH30 cells per membrane, consistent with near quantitative recovery of sarcoma cells spiked into whole blood. No CD45-negative, vimentin-positive cells were seen in the blood of any of the healthy controls.

### Kinetics of CTC in a mouse model of **Ewing** sarcoma metastasis

**Figure 2:**
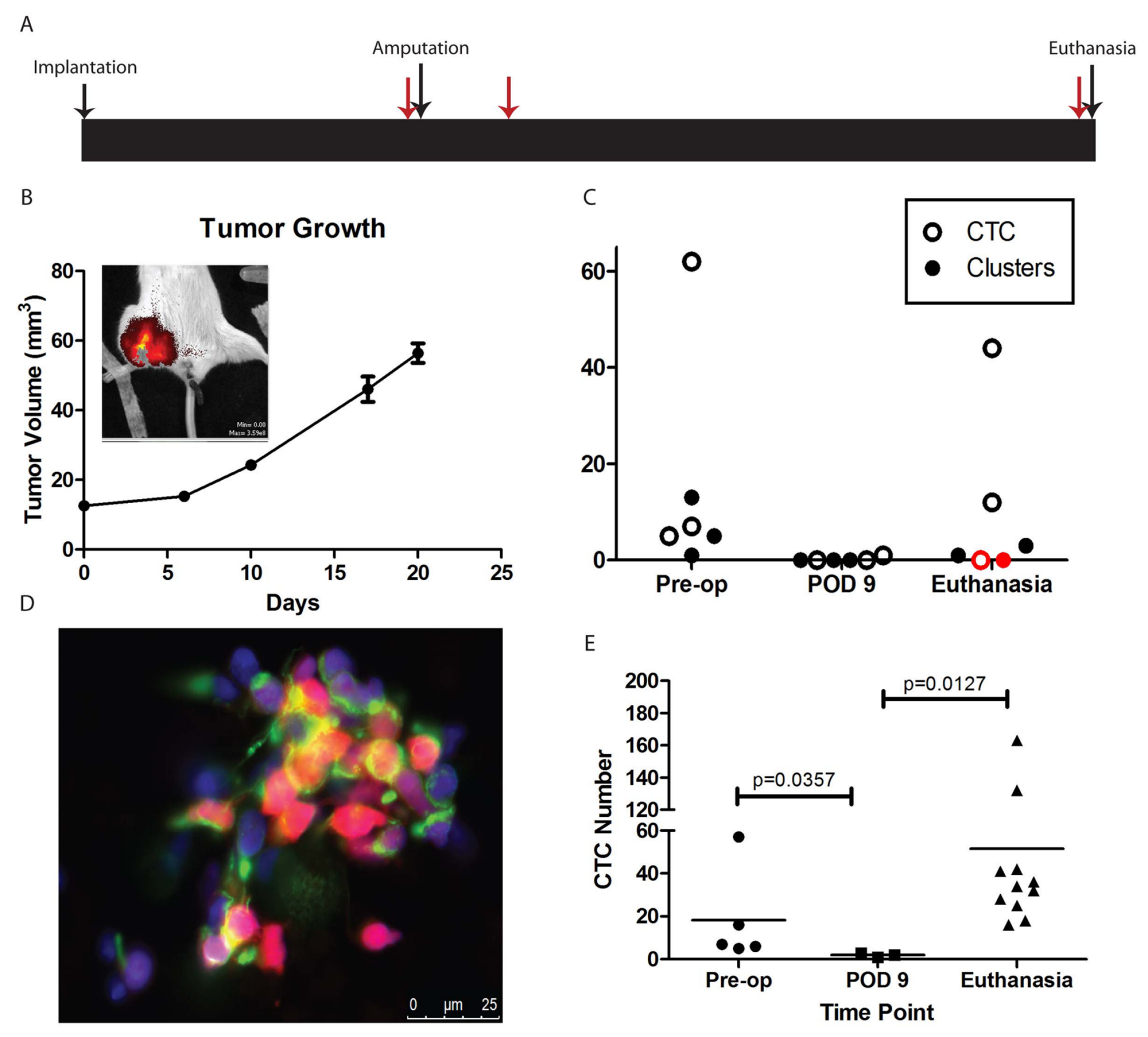
CTC in animal models **(A)** Schematic of experimental design. Major events (tumor implantation, hindlimb amputation, and euthanasia) are indicated with black arrows. Time points of blood draws are indicated with red arrows. **(B)** Growth curve of TC71 xenograft implanted in the pretibial space of NSG mice. Legs were amputated on Day 28, and mice were euthanized on either Day 35 or Day 56. Inset shows IVIS imaging to confirm retention of tdTomato in the growing tumor. **(C)** Numbers of CTC and CTC clusters recovered from each mouse. Note that the mouse with red symbols was found to have no evidence of disease on necropsy. **(D)** Representative image of a cluster of CTC recovered from one of the mice. Endogenous tdTomato is red, nuclei stained with DAPI are blue, and vimentin is green. **(E)** Quantification of CTC isolated from NSG mice bearing a TC32 xenograft. Mice were euthanized either at the time of amputation (“Pre-op”), on Day 9 after amputation (“POD 9”), or on Day 30 after amputation (“Euthanasia”), when subclinical metastases were expected to be present. Horizontal line represents the median value. Statistical analysis was performed using the Mann-Whitney test given deviation of the data from a normal distribution.

We next explored the ability of the CellSieve™ system to isolate and quantify CTC in a mouse model of Ewing sarcoma metastasis. A schematic of our experimental design is shown in Figure 2A. In a small pilot experiment, nine mice had a fragment of tdTomato-labelled TC71 tumor implanted in the pretibial space. When the maximal tumor diameter of the affected leg reached 15mm, all mice were imaged with the IVIS live animal imaging system which confirmed the retention of the TdTomato fluorescence in the xenografts (Figure 2B). Blood was collected by terminal blood collection from three mice, and affected limbs were amputated from the other six mice. Blood was collected from three mice one week after amputation, and from the remaining three mice four weeks after amputation. After microfiltration of the blood samples, the filters were immediately imaged. Both single tdTomato-positive cells and cell clusters were detected in blood samples from mice with large tumors (pre-amputation) and from mice with recurrent disease, but not in the blood samples from mice whose tumors had been amputated one week prior (Figure 2C). The tdTomato positive cells were also confirmed as vimentin positive and CD45 negative by immunofluorescence antibody staining (Figure 2D). Interestingly, the mouse without CTC at the time of euthanasia (red symbols in Figure 2C) also had no evidence of disease at necropsy, while the other two mice did (one local recurrence, one metastatic recurrence). To further validate these findings in a larger cohort of mice, nineteen mice were implanted with a fragment of the TC32 cell line derived xenograft in the pretibial space. When the maximal tumor diameter of the affected leg reached 15mm, five mice had blood collected terminally, and the remaining 14 were amputated. In the remaining cohort, blood was collected from three mice 9 days after amputation, and from the remaining eleven mice 30 days after amputation. Mice euthanized on post-op day (POD) 9 and on post-op day 30 were examined by necropsy. None of the mice euthanized on POD 9 had detectable tumor, but all of the mice euthanized on POD 30 were found to have metastatic disease. At each time point, the blood was collected and immediately filtered through the CellSieve™ filter, then stained for CD45 and vimentin prior to imaging. While CD45-negative, vimentin-positive sarcoma CTC nucleated cells were readily detected in mice with localized disease (all five mice euthanized prior to amputation) and in mice with metastatic relapse (all eleven mice which were euthanized on POD 30), there were substantially fewer CTC detected in the three mice which were evaluated on POD 9 (Figure 2E). Differences in cell number at each time point were statistically significant by the Mann-Whitney U test.

### Detection of CTCs and CTC clusters in sarcoma patients

**Figure 3:**
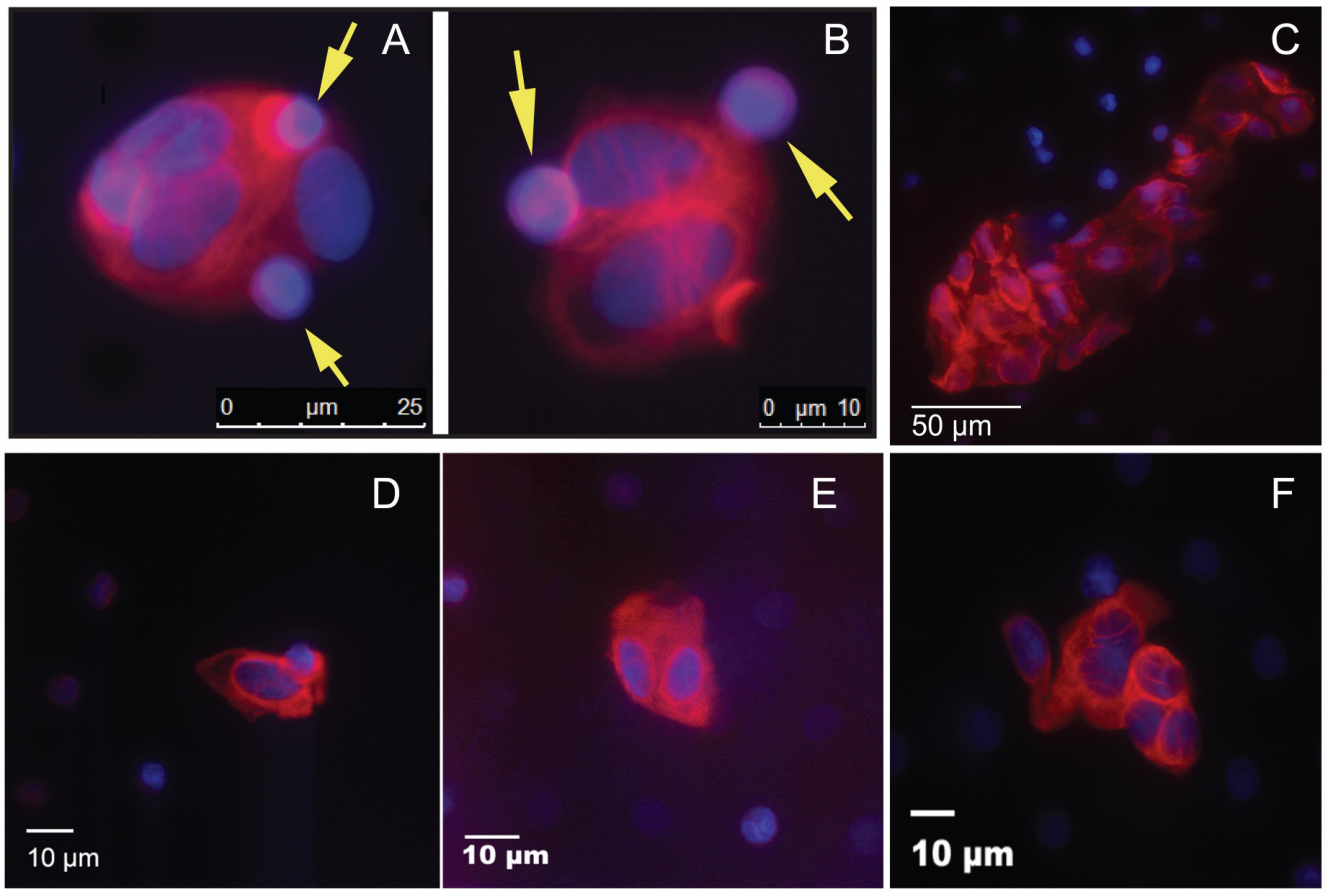
CTC from sarcoma patients CTC isolated from the blood of **(A** and **B)** a 38 year old man with a primary refractory localized paraspinal Ewing sarcoma, **(C)** a 17 year old girl with recurrent osteosarcoma, **(D)** a 3 year old boy with newly diagnosed alveolar rhabdomyosarcoma, **(E)** a 9 year old boy with metastatic embryonal rhabdomyosarcoma, and **(F)** a 23 year old man with localized malignant peripheral nerve sheath tumor. Vimentin is stained red, nuclei stained with DAPI are blue, and CD45 is stained purple. The smaller CD45+ lymphocytes (indicated by yellow arrows in panels A and B) are readily distinguished from larger CD45- sarcoma cells.

Our previous experiments validated the near quantitative recovery of sarcoma cells spiked into blood samples, and *in vivo* experiments with a mouse model of metastasis demonstrated that recovery of CTCs correlated with disease burden, so we decided next to determine whether we could identify CTCs in the blood of sarcoma patients. As proof of principle, we analyzed a total of 54 blood samples collected from 36 patients with different types of sarcoma (Table 1). Patient age ranged from 0 to 64 years, and the most common diagnoses included alveolar rhabdomyosarcoma, Ewing sarcoma, synovial sarcoma, and osteosarcoma. Twelve samples were drawn from patients with localized disease at the time of diagnosis, 8 were drawn from patients with localized disease undergoing treatment, 6 were from patients newly diagnosed with metastatic disease, and 3 were from patients with metastatic disease who were undergoing treatment. In addition, blood samples were assayed from 6 patients newly diagnosed with recurrent disease and 9 patients who had no radiographic evidence of disease. Circulating tumor cells were defined based on positive staining for vimentin, negative staining for CD45, and nuclear morphology distinct from that of normal white blood cells (Figure 3). CTCs were identified in 35 of the 54 samples (65%; range 0-514 cells/sample). Of the 19 samples with no detectable CTCs, 11 were collected at a time when there was radiographic evidence of tumor, and the remaining 8 CTC-negative samples were collected from patients with no radiographic evidence of active disease. In addition to single CTCs, we observed CTC clusters in blood samples obtained from 19 sarcoma patients (Figure 3). The CTC clusters contain multiple cellular nuclei, with irregular cell sizes and shapes, a high nucleus-to-cytoplasm ratio, are characterized by strong vimentin staining, and are CD45-negative.

**Table 1:** CTC detection from all samples

Pt #	Age	Dx	CTC	Clusters	Status
1a	38	ES	3	10	Refractory localized tumor
1b			1	1	Post-operative, radiographic NED
2	27	OS	0	44	Bilateral pulmonary metastases
3a	14	ES	11	16	Newly diagnosed localized tumor
3b			0	0	After neoadjuvant chemotherapy
3c			0	0	After resection of residual tumor, radiographic NED
3d			0	0	End of therapy, radiographic NED
4	45	DDLS	2	0	Recurrent retroperitoneal tumor
5	5	ES	0	0	End of therapy, radiographic NED
6a	9	ARMS	2	0	Newly diagnosed metastatic relapse
6b			0	0	End of therapy for relapsed disease, residual mass present
7	48	Poorly diff myxoid liposarcoma	2	1	Widely metastatic refractory disease
8	28	SS	0	0	Metastatic disease, after neoadjuvant chemotherapy
9a	60	ES	0	1	Newly diagnosed localized tumor
9b			0	0	After neoadjuvant chemotherapy
9c			0	0	After surgical resection, radiographic NED
9d			0	0	End of therapy, radiographic NED
10a	21	ES	16	0	Localized tumor, after neoadjuvant chemotherapy
10b			4	0	After resection of residual tumor, radiographic NED
10c			1	0	New metastasis
10d			1	0	Another new metastasis
11	23	ES	1	8	Newly diagnosed localized tumor
12a	15	OS	2	0	Newly diagnosed localized tumor
12b			0	0	End of therapy, radiographic NED
13	26	Epithelioid sarcoma	20	7	Widely metastatic recurrent disease
14a	31	SS	12	3	Localized tumor, after neoadjuvant chemotherapy
14b			1	0	After surgical resection, radiographic NED. Relapsed 2 months later
15	55	SS	0	0	Localized tumor, after neoadjuvant chemotherapy
16a	21	ES	1	0	Newly diagnosed metastatic disease
16b			0	0	After neoadjuvant chemotherapy
17	12	Myxoid liposarcoma	3	0	Newly diagnosed localized tumor
18	60	Chondrosarcoma	0	0	Newly diagnosed metastatic disease
19	58	Leiomyosarcoma	514	92	After neoadjuvant chemotherapy for relapsed disease
20a	16	OS	21	0	Newly diagnosed with metastatic relapse
20b			46	6	Third metastatic relapse
20c			6	1	After resection of metastatic disease
21	12	ES	8	3	Newly diagnosed localized tumor
22	23	OS	0	0	Localized tumor, after neoadjuvant chemotherapy
23	11	OS	16	2	Third metastatic relapse
24	32	SS	0	0	Newly diagnosed localized tumor, Day 2 of chemotherapy
25	52	SS	242	82	Newly diagnosed localized tumor
26a	14	ES	179	89	Newly diagnosed localized tumor
26b			508	26	After neoadjuvant chemotherapy
26c			0	0	End of therapy, radiographic NED
27	15	OS	0	0	Newly diagnosed with metastatic recurrence
28	53	OS	0	0	After resection of localized tumor, radiographic NED
29	29	DSRCT	0	0	Newly diagnosed with metastatic disease
30	<1	ERMS	26	3	Newly diagnosed with metastatic disease
31	18	SS	6	1	Newly diagnosed localized tumor
32	64	ARMS	2	0	Newly diagnosed with metastatic disease
33	23	MPNST	248	38	Newly diagnosed localized tumor
34	2	ARMS	446	28	Newly diagnosed localized tumor
35	9	ERMS	272	14	Newly diagnosed with metastatic disease
36	18	OS	3	0	Newly diagnosed localized tumor

ES = Ewing sarcoma; OS = osteosarcoma; DDLS = dedifferentiated liposarcoma; ARMS = alveolar rhabdomyosarcoma; ERMS = embryonal rhabdomyosarcoma; SS = synovial sarcoma; DSRCT = desmoplastic small round cell tumor; NED = no evidence of disease.

### Analysis of CTC in distinct subgroups of sarcoma patients

#### CTC are detectable at diagnosis in most sarcoma patients

**Figure 4:**
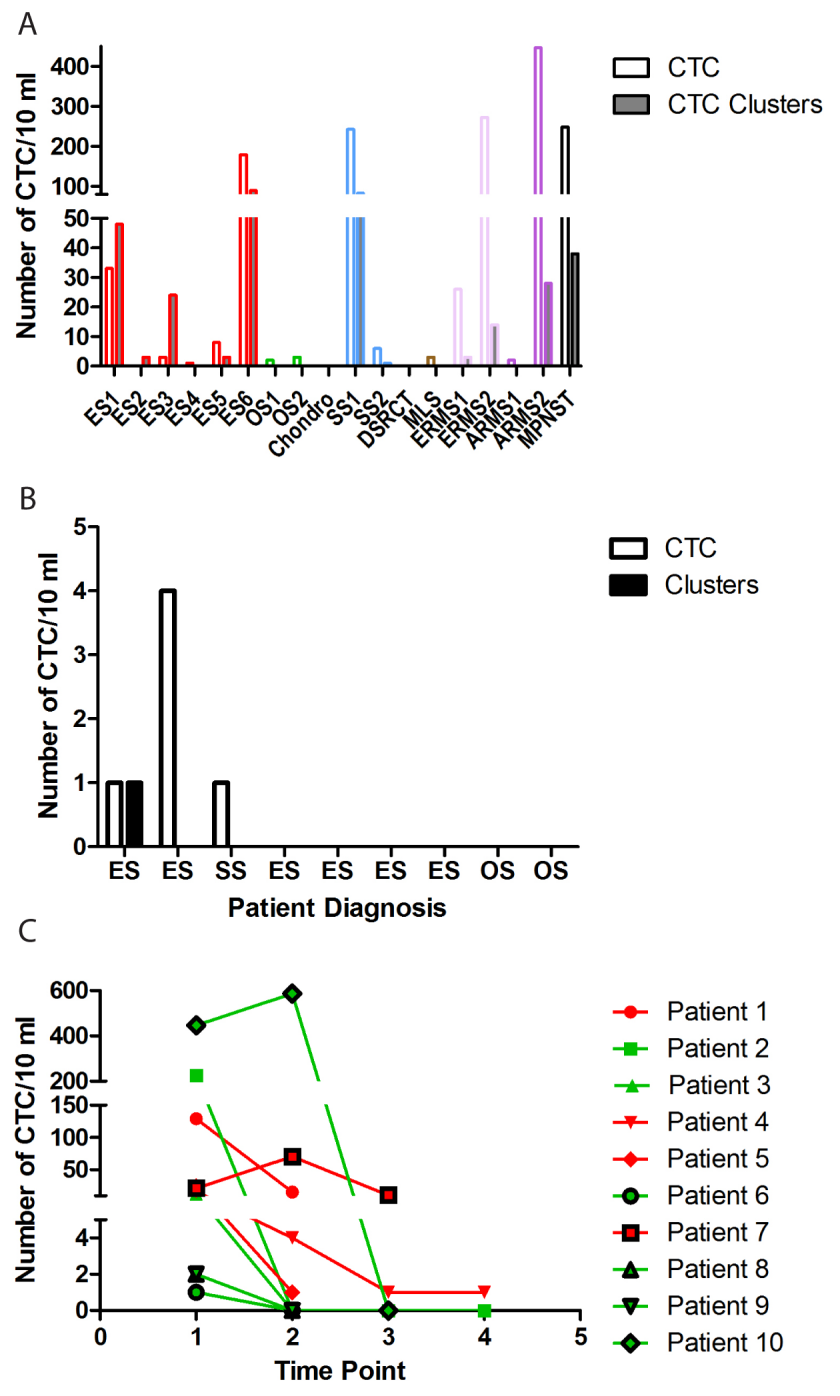
CTC are detected at diagnosis and decrease in response to therapy **(A)** CTC and CTC clusters were quantified from blood samples drawn from patients with the indicated diagnoses. ES = Ewing sarcoma, OS = osteosarcoma, Chondro = chondrosarcoma, SS = synovial sarcoma, DSRCT = desmoplastic small round cell tumor, MLS = myxoid liposarcoma, ERMS = embryonal rhabdomyosarcoma, and ARMS = alveolar rhabdomyosarcoma. **(B)** CTC and clusters were quantified from blood samples obtained from patients with the indicated diagnoses at a time when the patient had no radiographic evidence of disease. **(C)** CTC and CTC clusters were quantified from blood samples drawn from patients at various time points in their therapy. For graphing purposes, each cluster was considered to have 4 cells. Red lines and symbols represent patients who subsequently relapsed, and green lines and symbols represent patients who remain without radiographic evidence of disease.

We first sought to determine how frequently CTCs can be detected in the blood of newly diagnosed sarcoma patients. A total of 18 of the 54 samples were drawn from patients at the time of diagnosis. The diagnoses included Ewing sarcoma (6 patients), rhabdomyosarcoma (4 patients), and a variety of others (Figure 4A; Supplementary Table 1). CTC were detected in the blood of 16 of these patients (89%). The only exceptions were a patient with newly diagnosed, widely metastatic desmoplastic small round cell tumor and a patient with newly diagnosed metastatic chondrosarcoma. Interestingly, we noted a bimodal distribution of CTC within this group. Although 11 of the 18 patients had a relatively small number of cells detected (26 or fewer CTC and/or clusters), 5 patients had a substantial number of CTC and/or clusters (ranging from 179 to 446). These patients had diagnoses of Ewing sarcoma, malignant peripheral nerve sheath tumor, synovial sarcoma, embryonal rhabdomyosarcoma, and alveolar rhabdomyosarcoma. One of these patients (with embryonal rhabdomyosarcoma) had metastatic disease, but the remainder had localized disease. With these small numbers, there is no clear distinguishing characteristic between the patients with low CTC and those with high CTC.

#### CTC detection precedes clinical symptoms and radiographic detection of relapse

One important potential application of CTC quantification is the early detection of metastatic recurrence. We therefore investigated whether the presence of CTCs in the blood of patients with no radiographic evidence of disease correlates with impending relapse. Of the 54 samples analyzed, 11 were drawn from a total of 9 patients at a time when they had no radiographic evidence of disease (Figure 4B). Of these 9 patients, CTC were detected in 3. Each of those 3 patients relapsed within 1-2 months. Four of the patients whose samples had no CTC remain without evidence of disease (range 12 to 24 months). One patient was lost to follow up.

We also investigated dynamic changes in CTC numbers in response to treatment. Blood samples were collected at multiple time points from a total of 10 of the 36 patients (range 2-4 samples per patient), including 6 patients who were newly diagnosed, 3 who had just completed neoadjuvant chemotherapy, and 1 who had just suffered a relapse (Figure 4C). All of these patients had detectable CTC at the time of their initial blood draw, ranging from a low of 1 CTC to a high of 179 CTC and 89 clusters. All of these patients were treated after their initial blood draw. In 6 of these patients, CTC became undetectable after treatment. Five of these patients are currently in remission, with no radiographic evidence of disease (range 3-17 months). One had a radiographic complete response but is still being treated. Three of the 4 patients who had persistently detectable CTC relapsed (range 1-3 months). The other patient who had persistently detectable CTC is currently undergoing treatment, so is not evaluable.

### Molecular analysis of sarcoma CTCs

**Figure 5:**
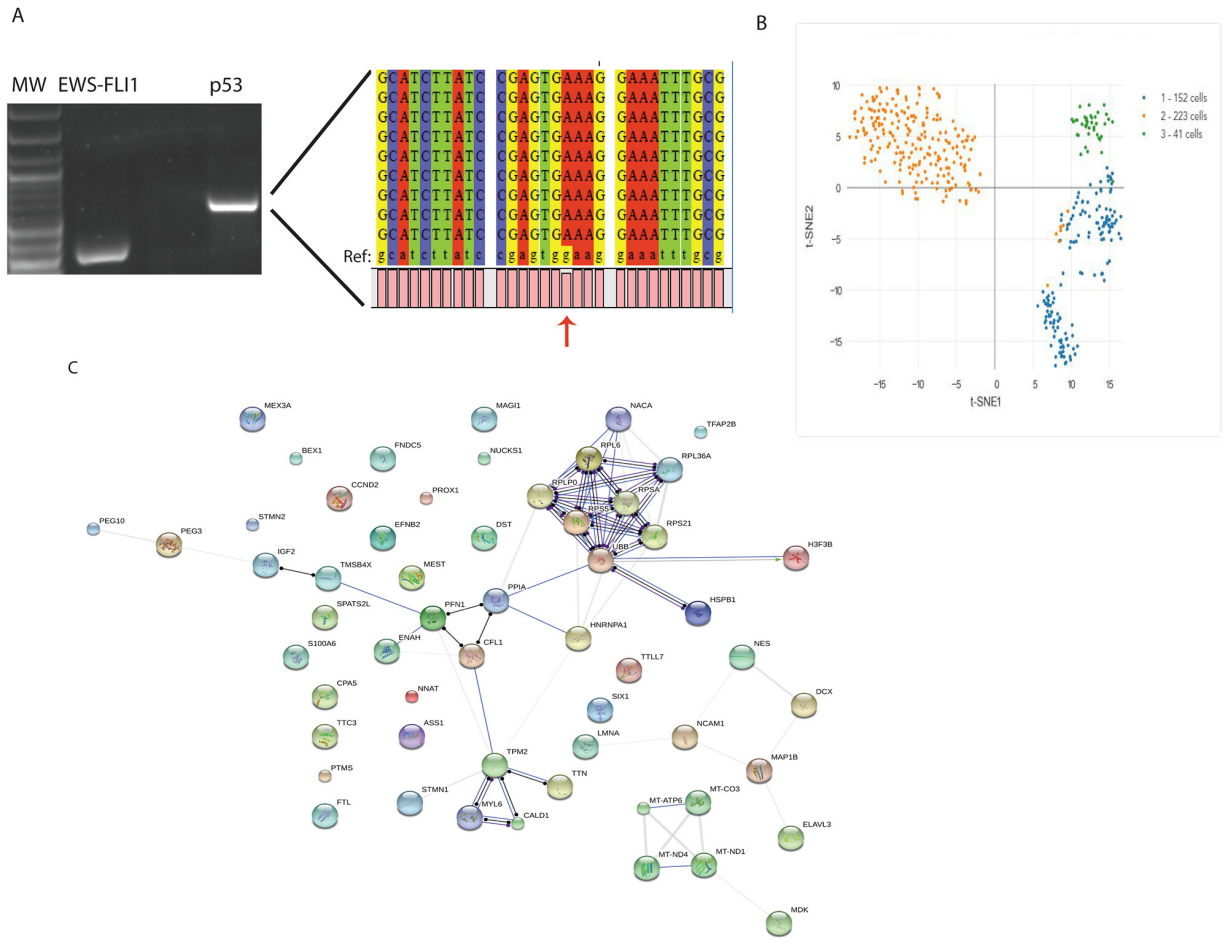
Molecular analysis of CTC from a Ewing sarcoma patient **(A)** RNA was extracted from CTC isolated from the blood of a patient with localized Ewing sarcoma of the thigh and amplified with primers specific for the EWS-FLI1 translocation (Lane 2) or p53 (Lane 4). Lane 1 contains a molecular weight ladder, and lane 3 is empty. The p53 amplicon was TA cloned, and the sequence obtained from each clone is shown, aligned with the reference sequence (indicated by Ref:). The mutant nucleotide, G592A, is identified by the arrow. **(B)** Individual CTC were analyzed on the 10x Genomics platform, and resultant gene expression profiles were clustered by the Cell Ranger program. A t-SNE projection is presented. Group 3 (green dots), containing 41 cells, is the CTC population. Group 1 (orange dots) is a cluster of red blood cells, and Group 2 (blue dots) represents contaminating white blood cells. **(C)** Protein interaction network of the protein-coding genes among the 70 genes differentially and strongly expressed in the CTC population.

One significant advantage of the CellSieve approach to collecting CTC, compared with flow cytometry-based approaches, is that the CTC are collected after minimal manipulation, offering the potential to analyze these cells at the molecular level for either diagnostic or research purposes. To demonstrate the feasibility of molecular analysis of the filter-captured CTCs, we used RT-PCR and Sanger sequencing to detect two genetic markers, the EWS-FL11 translocation and *TP53* mutation, in CTCs collected from both mouse and human patient blood samples. We extracted RNA from CTCs collected from the blood of a mouse implanted with an SK-ES-1 cell line xenograft and from CTCs collected from a human patient with Ewing sarcoma. An EWS-FLI1 fusion transcript was readily amplified from the mouse RNA (data not shown). The human RNA was evaluated by RT-PCR using primers for EWS-FLI1 and primers for p53. TP53 was chosen for this analysis because it is among the most frequently mutated genes in Ewing sarcoma [16]. Both primer sets amplified appropriately-sized amplicons (Figure 5A). The identity of the EWS-FLI1 amplicon was confirmed by Sanger sequencing. The p53 amplicon, generated using primers that flank a region known to be a mutational hotspot, was gel purified and Topo-TA cloned. A total of 9 individual clones were analyzed by Sanger sequencing. Each of these clones contained a previously described, but not previously suspected, point mutation (TP53 p.E198K/c.592G>A). This mutation has previously been reported in the COSMIC database in patients with non-small cell lung cancer, carcinoma of the esophagus, and malignant melanoma [17], but not in patients with Ewing sarcoma. It has a FATHMM score of 0.99, which is strongly suggestive of a deleterious mutation.

We also analyzed CTC from a patient with ARMS by single cell RNA-seq using the 10x Genomics platform. A total of 416 individual cells were sequenced. Analysis of the gene expression profile using t-distributed stochastic neighbor embedding [18] nonlinear dimensionality reduction (t-SNE), and unsupervised clustering revealed three distinct groups of cells which could be identified based on gene expression profile (Figure 5B). Group 1, containing 152 cells, was composed of white blood cells (based on expression of Class II HLA, IFITM2, IL1R2, LST2, and others) and Group 2, containing 223 cells, was composed of red blood cells (based on finding only hemoglobin transcripts in those cells). Group 3, containing 41 cells, contained the ARMS CTC based on gene expression profile. Based on these numbers, CTC represent approximately 10% of the cells retained by the CellSieve membrane. Among the top 150 genes most differentially expressed among the 3 groups, 70 were expressed at a significant level (≥1 UMI/cell) in the CTC population and were evaluated in detail (Supplementary Table 2). Among these genes are 28 that, either because they are part of a one of the previously published ARMS expression profiles or because they are not expressed in white blood cells but are expressed in either the myogenic lineage or in rhabdomyosarcoma cells, positively identify the cells in this cluster as ARMS CTC. These genes include MEG3, ASS1, SPATS2L, and DCX, all of which are part of an ARMS gene signature published by Davicioni and colleagues [19] ; MEST, IGF2, and PEG3, all identified as genes upregulated in ARMS by Rezvani and colleagues [20] ; and MAP1B, ENAH, CPA5, MAGI1, EFNB2, and PROX1, all of which contain PAX3/FKHR binding sites [21]. Although the patient had a known PAX3-FOXO1 translocation, this was not detected due to technical limitations of the 3’ priming and short-read sequencing that characterize the 10x Genomics platform. Interestingly, there were 5 genes identified which are typically imprinted, but since some are maternally expressed (such as MEG3) while others are paternally expressed (for example, PEG10), and many were identified as part of a study of hypomethylated genes that have lost imprinting in ARMS [22], supporting current hypotheses that epigenetic gene regulation plays a big part in ARMS biology. We also identified 23 genes (36% of those analyzed) that are associated with the cytoskeleton or have been directly implicated in cell-cell adhesion, migration or metastasis. The 70 genes expressed at a significant level in the CTC population demonstrate connectivity by STRING analysis (Figure 5C) and are enriched, by GO analysis, in proteins involved in a number of relevant biological processes, including those related to cell movement, protein complex organization, wound healing, protein translation, cell projections and cytoskeletal organization, and muscle filament assembly and sliding (Supplementary Table 3).

## DISCUSSION

The analyses presented in this pilot study demonstrate that we can reliably detect and collect sarcoma CTC with minimal manipulation of the retrieved cells, and provide proof of principle that this method can be used to monitor disease response, to identify patients in radiographic remission who are destined to relapse, as well as to study CTC biology. Using a low pressure, size-exclusive microfiltration system, we collected CTC from the blood of almost every patient with a newly diagnosed high grade sarcoma, correlated a decline in CTC number with response to therapy, and correlated the presence of CTC with impending relapse in patients with no radiographic evidence of disease. Although the number of patients was too small to draw statistically significant conclusions, these preliminary data demonstrate the potential of biomarkers of minimal residual disease in sarcomas to provide important prognostic information.

Although ours is not the first study of sarcoma CTC, our approach incorporates a novel technology that is applicable to all sarcoma histologies and enhances the ability to analyze collected cells to investigate their biology. Most previous studies of sarcoma CTC have been based on flow cytometry using a variety of immunophenotypic markers. These studies have been hampered by relatively poor sensitivity, and the use of putatively histology-specific markers (such as CD99 for Ewing sarcoma) not only may not increase specificity (since B lineage cells also express CD99) but limits general applicability of the technique, since many sarcoma histologies lack a specific cell surface marker (for example, osteosarcoma). Flow cytometry, by focusing on individual cells, eliminates cell clusters, but there is a growing literature suggesting that it is clusters of circulating tumor cells, rather than individual cells, that actually mediate metastasis [13]. Flow cytometry also cannot provide cellular morphologic information, which is critical in the identification and differentiation of tumor cell types.

Many currently used CTC platforms are based on a positive selection using antibodies against lineage-specific markers such as epithelial cytokeratins and EpCAM. CTC platforms based on epithelial markers are not suitable for the study of sarcoma samples because sarcoma tumor cells do not express epithelial markers. Our size-exclusion microfiltration system is an antibody-free approach, allowing capture of both epithelial and mesenchymal types of CTCs in blood samples with high sensitivity. By focusing on CD45 to eliminate normal white blood cells, vimentin to highlight sarcomas, and visual inspection of the entire cell, including the nucleus, we are able to accurately quantify CTC from a wide variety of sarcoma histologies. In addition, we can enumerate both individual cells and cell clusters, which may end up having a stronger predictive value for metastatic relapse. This is the first report of CTC clusters detected in human sarcoma patients, and they are more commonly seen in patients with newly diagnosed disease as well as in patients with metastases. The number of samples containing CTC clusters in our pilot study was too small to determine whether their presence has prognostic significance, as has been reported in breast cancer patients [13, 14], but this will be investigated in future work. Perhaps most importantly, our technique requires minimal processing of cells *ex vivo*, allowing us to perform sophisticated analyses (including single cell RNA-seq) on minimally manipulated cells. Our ability to collect CTC from mice is another strength of this approach, since robust preclinical models will allow biological studies to be performed that would be impossible in a clinical setting, given the rarity of these tumor types. Although the lack of a histology-specific marker may seem to be a weakness of this approach, we are confident that the cells we are analyzing are CTC. Not only are CTC readily distinguished phenotypically from normal white blood cells, but our molecular analyses clearly demonstrate that we have isolated tumor cells.

There are a number of potential clinical uses for CTC quantification in the care of sarcoma patients. A decrease in CTC number might be a more rapid indicator of response to therapy than imaging studies. In our animal model, the number of CTC increased with the growth of the primary tumor, decreased after amputation, and increased again in mice with metastatic relapse. The same trend was also observed in human sarcoma patient blood samples. Perhaps more importantly, the presence of CTC may distinguish patients with radiographically undetectable disease who are destined to relapse from those who are cured. In our mouse models, animals with no CTC were found to be metastasis-free on necropsy, while those with detectable CTC had metastases. Among our human sarcoma patients who had blood sampled at a time when there was no radiographic evidence of disease, those found to have CTC relapsed within months, while those without CTC remain disease-free for as long as 24 months. The ability to distinguish patients who are NED at the end of treatment but are destined to relapse from those who are cured would profoundly alter clinical practice. Clinical trials of maintenance therapy or other approaches to consolidation could be focused on those who require further therapy, sparing those already cured from unnecessary treatment and making results of such trials easier to interpret since the population would be more homogeneous.

Our approach will allow a detailed investigation of the molecular biology of metastasis. Because of the minimal manipulation required *ex vivo*, our method of isolating CTC is less likely than others to induce gene expression changes that will reflect the stress of the isolation procedure rather than reflecting *in vivo* biology. The ability to collect CTC clusters, in addition to individual CTC, will allow investigation of the role of these clusters in the metastatic process. Finally, the ability to isolate and analyze RNA from CTC has the potential to yield important new insights into the biology of sarcoma metastasis. Our initial single cell RNA-seq experiment demonstrated expression of a number of genes previously implicated in metastasis but never previously implicated in ARMS biology. A more detailed understanding of the biology of sarcoma metastasis, including comparisons between CTC and the primary tumor, will undoubtedly lead to new, more effective treatment approaches.

## MATERIALS AND METHODS

### *In vitro* validation

Ewing sarcoma cell lines TC71, TC32, and SK-ES-1 and alveolar rhabdomyosarcoma cell line RH30 were obtained from Dr. Jeffrey Toretsky (Georgetown University). Identity of the cell lines was confirmed by STR analysis in the Johns Hopkins University core facility. TC71 and RH30 cells were transduced with a tdTomato-encoding retroviral vector, pEF-1α-tdTomato, which was a kind gift from Dr. Venu Raman (Dept. Radiology, JHU). The successfully transduced cells were selected by puromycin resistance and FACS sorting as described [23]. To assess quantification of sarcoma cell recovery by the CellSieve™ filter, peripheral blood was drawn from 3 healthy volunteers in EDTA tubes, and tdTomato-labelled cells (counted by serial dilution) were spiked into the blood immediately prior to filtration.

### Animal experiments

NOD/SCID/IL-2Rγ-null (NSG) mice were implanted with 3 mm fragments of either a tdTomato-expressing TC71 xenograft or an unmanipulated TC32 or SK-ES-1 xenograft in the pre-tibial space as previously described [24]. The implanted limb was measured every 3-4 days. When the largest tumor diameter reached 15 mm, a cohort of mice was anesthetized with isofluorane and 0.4 ml of blood was collected terminally from the right ventricle after direct visualization of the heart by thoracotomy. The remaining mice had their affected limbs amputated as previously described [24]. From this remaining cohort, half of the mice were euthanized 1 week after amputation and 0.4 ml of blood was collected terminally, and the remaining mice were euthanized 4 weeks following amputation followed by terminal collection of 0.4 ml of blood from the right ventricle. Mice implanted with tdTomato-labelled TC71 xenografts were imaged using the Xenogen live animal imaging system to evaluate for metastatic disease prior to euthanasia. All animal experiments were approved by the Johns Hopkins University Animal Care and Use Committee. Results were analyzed for statistical significance by the Mann-Whitney test using Prism 6 software (GraphPad Software, Inc., La Jolla, CA).

### Patient samples

Blood samples were collected under a protocol approved by the Johns Hopkins University Institutional Review Board, after obtaining informed consent. A total of 54 blood samples were collected from 36 patients who presented to Johns Hopkins University Sidney Kimmel Comprehensive Cancer Center with a diagnosis of high-grade sarcoma between February 2015 and November 2016. A total of 10 ml of blood was collected in either EDTA blood collection tubes or CellSave tubes (Janssen Diagnostics, Raritan, NJ) and was processed for CTC detection within 2 hours of collection in all cases.

### Circulating tumor cell detection

Microfiltration of whole blood was performed using the CellSieve™ microfiltration system and reagent kits as previously described [15]. Briefly, blood was diluted 1:1 with prefixation buffer and incubated at room temperature for 15 min. The prefixed blood sample was filtered through CellSieve^™^ filter at a flow rate of 5 mL per minute. The filter was washed with phosphate-buffered saline (PBS) to clean up normal blood residue and then incubated with postfixation buffer for 20 min. The postfixation buffer was removed and the filter was washed with PBS prior to incubation with permeabilization buffer at room temperature for 20 min. The filter was then washed with PBS and incubated with a fluorescent-labeled antibody cocktail containing anti-vimentin antibody (EF570) and anti-CD45 antibody (Cyanine 5) at room temperature for 1 hour followed by sequential washes with PBS-T and PBS. The filter was mounted on a slide using mounting solution with 4’,6-diamidino-2-phenylindole (DAPI) and a cover glass. The cell morphology and antibody staining patterns on the filter were examined with an Olympus IX81 motorized inverted microscope with a Hamamatsu C9100-02 front-illuminated EM-CCD camera. A CTC was defined as vimentin-positive, CD45-negative and with nuclear morphology characteristic of malignant cells.

### Molecular analysis of circulating tumor cells

After filtration of blood through the CellSieve^™^ microfiltration system, cells were directly lysed on the membrane for RNA collection. RNA was extracted using the RNeasy Micro Kit (Qiagen, Valencia, CA) according to manufacturer’s protocol, then amplified using the SMARTer RACE cDNA Amplification Kit (Takara Bio USA, Madison, WI). Resulting cDNA was used for PCR amplification to specifically detect EWS-FLI1 or p53 mRNA using primers listed in Supplementary Table 4 with the following conditions: initial denaturation at 95°C for 3 minutes followed by 40 cycles of 95°C for 20 seconds, 53°C for 20 seconds, and 72°C for 1 minute. The single PCR product was purified using a QIAquick PCR purification kit (Qiagen), cloned into the pCR4-TOPO TA vector using a TA cloning kit (Invitrogen) according to manufacturer’s recommendations, and then 9 individual clones were sequenced on an Applied Biosystems 3730xl DNA Analyzer.

Single cell RNA-seq analysis was performed in conjunction with the Johns Hopkins Genetic Resources Core Facility using the 10x Genomics (San Francisco, CA) platform. For this study, cells were backwashed from the CellSieve^™^ membrane and red blood cells were removed from the resultant cell suspension by Ficoll gradient centrifugation. These semi-purified CTC were combined with the Chromium bead-attached primer library, emulsification oil and DNA polymerase on a GemCode Chip. The GEM beads were then thermocycled, the emulsion broken and DNA recovered according to the 10X Genomics protocol. Libraries were prepared by Covaris shearing to form ∼800 bp molecules, end repaired, A-tailed and ligated with adapter as detailed in the protocol. Following SPRI cleanup, libraries were quantified by qPCR (KAPA) and their size assayed by Bioanalyzer (Agilent) electrophoresis. Resulting libraries were sequenced on an Illumina HiSeq2500, generating 571,000 mean reads per cell for 416 cells. Gene expression data was analyzed using the Cell Ranger software provided by 10x Genomics. Results were annotated with GO pathway and functional analysis, and protein interactions surveyed using STRING networking tools v 10.0 [25].

## SUPPLEMENTARY MATERIALS TABLES


