# Supplementary material for: Size-based detection of sarcoma circulating tumor cells and cell clusters
Source: Oncotarget. 2017 Aug 24;8(45):78965–77. doi: 10.18632/oncotarget.20697 (PMC5668012; doi:10.18632/oncotarget.20697)
Supplement: Supplementary file 1 [file oncotarget-08-78965-s001.pdf]

Size-based detection of sarcoma circulating tumor cells and cell clusters

SUPPLEMENTARY MATERIALS

REFERENCES

1. Jin Z, Lahat G, Korchin B, Nguyen T, Zhu QS, Wang X, Lazar AJ, Trent J, Pollock RE, Lev D. Midkine enhances soft-tissue sarcoma growth: a possible novel therapeutic target. Clin Cancer Res. 2008; 14:5033-5042.
2. Rouillard AD, Gundersen GW, Fernandez NF, Wang Z, Monteiro CD, McDermott MG, Ma'ayan A. The harmonizome: a collection of processed datasets gathered to serve and mine knowledge about genes and proteins. Database (Oxford). 2016; 2016.
3. Gluer S, Schelp C, von Schweinitz D, Gerardy-Schahn R. Polysialylated neural cell adhesion molecule in childhood rhabdomyosarcoma. Pediatr Res. 1998; 43:145-147.
4. Yu Y, Davicioni E, Triche TJ, Merlino G. The homeoprotein six1 transcriptionally activates multiple protumorigenic genes but requires ezrin to promote metastasis. Cancer Res. 2006; 66:1982-1989.
5. Sana J, Zambo I, Skoda J, Neradil J, Chlapek P, Hermanova M, Mudry P, Vasikova A, Zitterbart K, Hampl A, Sterba J, Veselska R. CD133 expression and identification of CD133/nestin positive cells in rhabdomyosarcomas and rhabdomyosarcoma cell lines. Anal Cell Pathol (Amst). 2011; 34303-318.
6. Chastre E, Abdessamad M, Kruglov A, Bruyneel E, Bracke M, Di Gioia Y, Beckerle MC, van Roy F, Kotelevets L. TRIP6, a novel molecular partner of the MAGI-1 scaffolding molecule, promotes invasiveness. FASEB J. 2009; 23916-928.
7. Liu Y, Zhang JB, Qin Y, Wang W, Wei L, Teng Y, Guo L, Zhang B, Lin Z, Liu J, Ren ZG, Ye QH, Xie Y. PROX1 promotes hepatocellular carcinoma metastasis by way of up-regulating hypoxia-inducible factor 1alpha expression and protein stability. Hepatology. 2013; 58:692-705.

Supplementary Table 1: Clinical characteristics of patients evaluated at diagnosis

Diagnosis	Disease Status	CTC	CTC Clusters
ARMS	Metasatic (bone)	2	0
ARMS	Localized	446	28
ERMS	Metastatic (peritoneum)	26	3
ERMS	Metastatic (lungs)	272	14
Ewing sarcoma	Localized	11	16
Ewing sarcoma	Localized	0	1
Ewing sarcoma	Localized	1	8
Ewing sarcoma	Metastatic (lungs)	1	0
Ewing sarcoma	Localized	8	3
Ewing sarcoma	Localized	179	89
MPNST	Localized	248	38
Myxoid liposarcoma	Localized	3	0
Chondrosarcoma	Metastatic	0	0
DSRCT	Widely metastatic	0	0
Osteosarcoma	Localized	2	0
Osteosarcoma	Localized	3	0
Synovial sarcoma	Localized	242	82
Synovial sarcoma	Localized	6	1

Supplementary Table 2: Genes upregulated in ARMS CTC that are involved in cell-cell adhesion, cytoskeletal dynamics, migration, or metastasis

Gene	Description
MDK	Secreted growth factor. ALK ligand. Promotes migration/proliferation/angiogenesis. Implicated in RMS by Jin et al. [1]
STMN1	Destabilizes microtubules
MAP1B	Associated with neurite outgrowth, membrane blebbing, and intracellular vesicular transport; Has a PAX3/FKHR binding site in the promoter [2]
DST	Anchors intermediate filaments to the actin cytoskeleton; integrin binding.
CALD1	Regulates actin/myosin interactions in smooth muscle;
NCAM1	Regulates cell/cell adhesion; expressed in RMS [3]
LMNA	Intermediate filament protein, component of the nuclear lamina
ENAH	Role in cytoskeletal remodeling/dynamics, especially lamellipodial and filopodial dynamics in migrating cells
DCX	Microtubule binding protein involved in neuronal migration;
SIX1	Required for normal development of migratory myogenic progenitor cells and promotes metastasis in a mouse model of RMS [4]
STMN2	Destabilizes microtubules
NES	Intermediate filament protein; Marker of neuronal stem cells; Co-expression of Nestin and CD133 marks cancer stem cells, including in RMS [5]
MAGI1	Important for regulating cell-cell contacts; Has a PAX3/FKHR binding site in the promoter [2]; May be involved in cell invasiveness [6]
EFNB2	Transmembrane signaling protein; crucial for migration and adhesion;
TTLL7	Regulates microtubule dynamics
PROX1	Specifically implicated in metastasis [7]
TPM2	Actin-binding protein
RPSA	High affinity non-integrin laminin binding protein/receptor implicated in metastasis
MALAT1	lncRNA transcriptional regulator; implicated in metastasis;
TMSB10	Actin binding protein that sequesters G actin;
MYL6	Myosin light chain 6
PFN1	Actin binding protein regulated by extracellular signals;
CFL1	Regulates actin polymerization; found in invadopodia and lamellipodia; downstream of RhoA and ROCK

Supplementary Table 3: GO Analysis of Biological Processes

Pathway Description	Count	False Discovery Rate
Movement of cell or subcellular component	16	0.000227
Protein complex subunit organization	16	0.000453
Response to wounding	11	0.00196
Translational termination	6	0.00343
Cell projection morphogenesis	10	0.0067
Cytoskeletal organization	10	0.0111
Actin filament-based process	7	0.0196
Skeletal myofibril assembly	2	0.0198
Muscle filament sliding	3	0.0248

Supplementary Table 4: PCR Primers

Primer	Sequence
P53 primer F	5’-tctgtgacttgcacgtactc-3’
P53 primer R	5’-cacggatctgaagggtgaaaa-3’
EWS ESBP1	5’- cgactagttatgatcagagcagt-3’
Fli1 ESBP2	5’- ccgttgctctgtattcttactga-3’
